# Supplementary material for: Influence of artificial intelligence in education on adolescents’ social adaptability: The mediatory role of social support
Source: PLoS One. 2023 Mar 17;18(3):e0283170. doi: 10.1371/journal.pone.0283170 (PMC10022785; doi:10.1371/journal.pone.0283170)
Supplement: Attachment — Submitted filename: Response to Reviewers.docx [file pone.0283170.s002.docx]

**Responds to journal requirements**

1. Please ensure that your manuscript meets PLOS ONE's style requirements, including those for file naming.

**Respond 1**: We have comleted the modification according to the PLOS ONE style tempates, and the manuscript might meet the PLOS ONE’s style requirements. Please see our revised manuscript.

2.During your revisions, please note that a simple title correction is required: "Adolescent's" should be replaced by "Adolescents' ". Please ensure this is updated in the manuscript file and the online submission information.

**Respond 2:** The “Adolescent’s” has been replaced by “Adolescents’” in the manuscript file and the online submission information.

3.please ensure that you provide the correct grant numbers for the awards you received for your study in the ‘Funding Information’ section.

**Respone 3:** We have provided the correct grant number (No:2020zd003) for the awards we received for our study in the “Funding Information” section in the revised cover letter.

4. Thank you for stating the following financial disclosure, at this time, please address the following queries.

**Respone 4:** We have amended the funding disclosure in the cover letter as following: This study received support from The Major Project of Guangzhou Educational Science Planning (No: 2020zd003). The funders had no role in study design, data collection and analysis, decision to publish, or preparation of the manuscrip.

5. Please amend either the title on the online submission form (via Edit Submission) or the title in the manuscript so that they are identical.

**Respone 5:** We have amended the title on online submission form and the title in the manuscript, they are already identical now.

6.Please ensure that you have an ORCID iD and that it is validated in Editorial Manager.

**Respone 6:** I, as a corresponding auther, have been authenticated the pre-existing iD in Editorial Manager.

7.PLOS only allows data to be available upon request if there are legal or ethical restrictions on sharing data publicly.

**Respone 7:** We have addressed the following prompts in the revised cover letter:

All relevant data are in the paper and its Supporting Information files.

8. Please review your reference list to ensure that it is complete and correct.

**Respone 8.** We have reviewed my reference list again, and it is complete and correct.

**Responds to reviewer#1**

1 - Only main effects and mediations are mentioned. Please also test the effects by social sttaus and gender/sex.

**Respond 1:** In this study, we mainly consider to explore the impact of AIEd on adolescents’ social adaptability, and the mediatory role of social support between AIEd and social adaptability. Of course, gender, social status, race, and all, that you mentioned, may be influencing factors, but we have not included them in this study. We will consider the role of these factors in our future research. Thank you for your review and suggestions.

2- Ple ase add data on fit statistics.

**Respond 2：**we add data on fit statistics in the revision manuscript as follow:

We tested the mediatory model of parent-chlid relationship between AIEd and social adaptability, and the parent-child communication between AIEd and social adaptability, the mediatory moedel showed an excellent fit to the data: χ^2^/df = 4.81, comparative fit index (CFI) = 1.00, root mean square error of approximation (RMSEA)= 0.019; χ^2^/df = 3.92, comparative fit index (CFI) =1.00, root mean square error of approximation (RMSEA)= 0.023.

3- Please report standardized coefficients (rather than unstandardized b) in the paper and also in figures.

**Respond 3:** In our manusript, *β* (Beta) in the paper is the standardized coefficients rather than understandardized coefficients B*.* and we re-report the standardizd coefficients in the figures*.*

4- What is data on ethnicity / social class?

**Respond 4:** Thank you for your review and suggestion. In this study, we did not consider the impact of ethnicity/social class, so we did not collect those data. In our future study, we will collect the ethnicity/social class data, and consider their impact.

**Responds to reviewer#2**

In Abstract: Please be consistent, authors used AIed and AIEd variations. Moreover, please use full words instead of abbreviations for the very first time.

**Respond:** We are sorry for the misspelling. We have replaced AIed with AIEd, and they are identical now. Besides, as your suggestion, we have used full words instead of abbreviations for the very first time.

While applying ANOVA, how the the problem was formulated for non-AI group? Does AI usage questionnaires represent the AI group and rest parent-child relationship scholar or teacher student scales lies in non-AI group?
**Respond:** Although subjects in our study are students come from Artificial Intelligence Curriculum Reform Experimental Schools in Guangzhou, but not all students use artificial intelligence for learning. So, the AI usege questionnaires we used in study has one items: “*Are you using AI or smart phones, tablets and other intelligent devices to learning*”, is scored with options 1= *yes* and 2 = *no*. Based on their response, students were divided into the artificial intelligence group and non- artificial intelligence group, 1 is AI group and 2 is non-AI group. The parent-child relationship scale and the teacher-student relationship scale are filled in by all students participating in the survey, whether they use artificial intelligence for learning or not. All subjects completed all questionnaires.

Did authors try other methods then ANOVA? If not, then why ANOVA was chosen, please elaborate.

**Respond:** We also carried out average value analysis, and the result showed that they were consistent with the result in manuscript, it is significant. Because ANOVA can determine the influence of controllable factors on the research results, so we choose present the result of ANOVA in manuscript.
